# Results of the Implementation of a Pilot Model for the Bidirectional Screening and Joint Management of Patients with Pulmonary Tuberculosis and Diabetes Mellitus in Mexico

**DOI:** 10.1371/journal.pone.0106961

**Published:** 2014-09-17

**Authors:** Martín Castellanos-Joya, Guadalupe Delgado-Sánchez, Leticia Ferreyra-Reyes, Pablo Cruz-Hervert, Elizabeth Ferreira-Guerrero, Gabriela Ortiz-Solís, Mirtha Irene Jiménez, Leslie Lorena Salazar, Rogelio Montero-Campos, Norma Mongua-Rodríguez, Renata Baez-Saldaña, Miriam Bobadilla-del-Valle, Jesús Felipe González-Roldán, Alfredo Ponce-de-León, José Sifuentes-Osornio, Lourdes García-García

**Affiliations:** 1 Dirección del Programa de Micobacteriosis, Centro Nacional de Programas Preventivos y Control de Enfermedades, México, Distrito Federal, México; 2 Centro de Investigación sobre Enfermedades Infecciosas, Instituto Nacional de Salud Pública, Cuernavaca, Morelos, México; 3 Dirección del Programa de Salud en el Adulto y en el Anciano, Centro Nacional de Programas Preventivos y Control de Enfermedades, México, Distrito Federal, México; 4 Laboratorio de Microbiología, Instituto Nacional de Ciencias Médicas y de Nutrición “Salvador Zubirán”, México, Distrito Federal, México; 5 Dirección General, Centro Nacional de Programas Preventivos y Control de Enfermedades, México, Distrito Federal, México; 6 Dirección Médica, Instituto Nacional de Ciencias Médicas y de Nutrición “Salvador Zubirán”, México, Distrito Federal, México; Centers for Disease Control, Taiwan

## Abstract

**Background:**

Recently, the World Health Organisation and the International Union Against Tuberculosis and Lung Disease published a Collaborative Framework for the Care and Control of Tuberculosis (TB) and Diabetes (DM) (CFTB/DM) proposing bidirectional screening and joint management.

**Objective:**

To evaluate the feasibility and effectiveness of the CFTB/DM in Mexico. **Design**. Prospective observational cohort. **Setting**. 15 primary care units in 5 states in Mexico. **Participants**: Patients aged ≥20 years diagnosed with DM or pulmonary TB who sought care at participating clinics. **Intervention**: The WHO/Union CFTB/DM was adapted and implemented according to official Mexican guidelines. We recruited participants from July 2012 to April 2013 and followed up until March 2014. Bidirectional screening was performed. Patients diagnosed with TB and DM were invited to receive TB treatment under joint management. **Main outcome measures**. Diagnoses of TB among DM, of DM among TB, and treatment outcomes among patients with DM and TB.

**Results:**

Of 783 DM patients, 11 (1.4%) were unaware of their TB. Of 361 TB patients, 16 (4.4%) were unaware of their DM. 95 TB/DM patients accepted to be treated under joint management, of whom 85 (89.5%) successfully completed treatment. Multiple linear regression analysis with change in HbA1c and random capillary glucose as dependent variables revealed significant decrease with time (regression coefficients (β)  = −0.660, (95% confidence interval (CI), −0.96 to −0.35); and β = −1.889 (95% CI, −2.77 to −1.01, respectively)) adjusting by sex, age and having been treated for a previous TB episode. Patients treated under joint management were more likely to experience treatment success than patients treated under routine DM and TB programs as compared to historical (adjusted OR (aOR), 2.8, 95%CI 1.28–6.13) and same period (aOR 2.37, 95% CI 1.13–4.96) comparison groups.

**Conclusions:**

Joint management of TB and DM is feasible and appears to improve clinical outcomes.

## Introduction

Tuberculosis (TB) remains a major cause of morbidity and mortality in low- and middle-income countries, where the numbers of individuals with type 2 diabetes mellitus (DM) are also increasing rapidly. [1], [2] Many studies have explored the relationship between DM and TB, including a recent systematic review in which the risk of TB in DM patients was shown to be three-fold higher than that of individuals without DM. [3] Moreover, the available evidence indicates that DM comorbidity worsens the clinical outcomes of TB patients. [4], [5]

Recently, the World Health Organisation (WHO) and the International Union against Tuberculosis and Lung Disease (Union) recognised the need for international guidelines regarding the joint management of TB and DM and published a provisional Collaborative Framework for the Care and Control (CFTB/DM) of both diseases. [6] This framework emphasized establishment of mechanisms of collaboration between national programs of TB and DM, bidirectional screening of TB and DM, and integration of TB and DM management. Integration of services in low and middle resource settings has long been debated. A recent systematic review found limited evidence of its effectiveness in improving health outcomes, and some evidence to suggest that it may improve efficiency but may not be appropriate in all circumstances. [7] Bidirectional screening of TB and DM has recently been reviewed. [8] Results showed that TB prevalence among patients with DM is high, ranging from 1.7% to 36%, and increasing with rising TB prevalence in the underlying population as well as with DM severity. Screening patients with TB for DM also yielded high prevalence of DM ranging from 1.9% to 35%. More recently, the WHO/Union framework has been tested in pilot experiences which demonstrated the feasibility of bidirectional screening. [9], [10] To our knowledge, there is no published literature on integration of diabetes and tuberculosis management.

We have previously documented that the high prevalence of DM in Mexico results in a considerable proportion of TB cases that are attributable to this disease. [11] Thereby, we conducted this study in order to evaluate the feasibility and effectiveness of bidirectional screening and joint management of TB and DM as recommended by WHO/Union.

## Methods

We conducted an observational cohort study recruiting participants from July 2012 to April 2013. Patients diagnosed with DM and TB were followed up until March 2014. We adapted the CFTB/DM, proposed by WHO and Union, according to the current official Mexican standards for the management of TB and DM. [6], [12], [13] Joint management focused to link DM and TB programs at the level of service delivery. It aimed: 1) to bring together TB and DM screening and health services through evaluation and testing of TB and DM among patients with DM and TB respectively, conducted by trained nurses to consenting patients and 2) periodical monitoring of glucose levels, provision of referral to specialised outpatient clinics in case of difficult control and counselling sessions to patients and their families by nurses providing directly observed tuberculosis therapy (DOTS). No additional staff was recruited. The program differed from the usual practice in that neither screening of TB was offered to DM patients nor screening of DM was offered to TB patients. Patients with DM and TB had their glucose level monitored monthly while under TB treatment. To implement the pilot joint management program we conducted the following: 1) invitation of the federal TB and DM programs to state and local levels to participate; 2) establishment of mechanisms for inter-programmatic collaborations with emphasis placed on the participation of state and local levels in planning of activities; 3) cascade training of health personnel who would be participating in screening and joint management of patients; 4) invitation to TB and DM patients and their families to participate; 5) weekly electronic submission and review of case report forms from the health jurisdiction to the federal level; 6) monthly monitor visits to participating primary care clinics; and 7) final meeting to present the results.

### Study sites and study population

Fifteen publicly managed primary care centres in 5 health jurisdictions in northern and central Mexico were selected based on the highest burdens of TB/DM comorbidity in 2011; their availability of infrastructure for detection, screening and treatment of TB/DM and the willingness of health authorities to participate. We recruited individuals of both sexes who were ≥20 years of age, had received a previous diagnosis of DM or pulmonary TB and who attended the participating clinics during the study period.

### Detection of TB in DM patients

Individuals who had received a previous diagnosis of DM by a physician, were administered a structured questionnaire investigating epidemiological or clinical information suggesting active TB. Among individuals with one or more of these conditions, acid-fast bacilli (AFB) test was performed in three sputum samples. Among those testing positive, *Mycobacterium tuberculosis* culture was performed. Individuals with suspected extrapulmonary disease were referred to specialised care.

### Detection of DM in TB patients

Among patients with confirmed pulmonary TB (AFB in sputum smears or *Mycobacterium tuberculosis* in cultures), a structured questionnaire was used to investigate data suggestive of DM. Regardless of results, random capillary blood glucose, and HbA1c and fasting glucose levels were measured in venous blood.

### Joint management of DM and TB

Patients with confirmed pulmonary TB/DM diagnoses, who were ≥20 years of age, who were residents in the study area and who were able to receive TB treatment in a participating primary care clinic were invited to participate under joint management according to DM and TB official guidelines. [12], [13] If consenting, they were referred to participating primary care units. Patients were treated under DOTS (directly observed treatment, short course) strategy using the WHO standard regimen in which therapy was initiated with 4 drugs (2HRZE/4HR) for newly diagnosed patients and 5 drugs (2HRZES/1HRZE/5HRE) for previously treated patients all given under direct observation of treatment at the clinics. Patients harbouring isolates resistant to both isoniazid and rifampin were treated with a second-line standardised regime of at least 4 drugs that were highly likely to remain effective for 18–24 months after culture conversion.

Weekly random capillary blood glucose, monthly fasting glucose and quarterly HbA1c measurements were performed. Capillary punctures and venous blood draws were conducted by personnel providing TB treatment. Blood samples were processed in the laboratories of participating clinics. On detection of abnormal results, patients were informed of the results and referred to outpatient specialised clinics (UNEME, EC, for its acronym in Spanish) where glucose control followed standard treatment guidelines. [14]

Counselling sessions were weekly conducted by personnel providing TB treatment and addressed adherence to TB and DM treatment regimens, daily physical exercise program, attention to diet and importance of family involvement.

### Mycobacteriology

Sputum samples were processed for acid fast bacilli smears and *M. tuberculosis* culture and drug susceptibility tests according to standardised procedures. [15] The Institute of Diagnosis and Epidemiological Reference performed quality control analyses for all participating laboratories.

### Diagnosis of DM

The diagnosis of DM among TB patients was based on the following criteria: a) fasting glucose level ≥126 mg/dL and b) HbA1c level ≥6.5%. [16]

### Statistical Analysis

The primary outcomes were TB testing rate amongst new and prevalent DM clients, DM testing among new TB patients and treatment outcomes amongst patients treated under joint management. Laboratory and data processing personnel were blinded to the study. We used WHO definitions of treatment outcomes except default which was defined according to the Mexican official guidelines. [12], [17] Briefly, failure was defined when AFB microscopies or cultures were positive at five months or later during treatment. Cure was defined when treatment was completed with the disappearance of signs and symptoms with two or more acid-fast bacilli smears or cultures with negative results at the end of therapy. Treatment completion was defined when a patient completed treatment but did not meet the criteria to be classified as a cure or a failure. Death was defined when a patient died of any cause during therapy. Treatment success was defined by the the sum of patients who were cured and those who had completed treatment. Mexican guidelines have defined default when a patient interrupts treatment for 30 days or more rather than 60 days defined by WHO so as to be able to timely prevent that patients drop out from treatment. Incomplete and inaccurate case report forms (including missing patient identifiers, missing or inconsistent testing information or missing or inconsistent treatment outcomes) were queried and corrected by clinic staff through checking clinical and laboratory records. The sociodemographic, epidemiological and clinical data of patients who were screened for DM and TB were analysed according to the screening results. Among patients with both DM and TB, characteristics were compared between patients with treatment success versus those who defaulted, failed, died or were transferred out.

Using robust random effects linear regression for longitudinal data, we estimated regression coefficients (β) and 95% confidence intervals (CI), to determine if HbA1c, serum glucose and random capillary glucose changed significantly during treatment adjusting for sex, age, and previous TB treatments. The variables included in the models were those with *p*-values ≤0.20 in bivariate analysis or with biological plausibility. Covariates were arrived to by using hierarchical backward elimination approach. We tested the models with Hausman and Breusch-Pagan tests.

We used two comparison groups to evaluate whether joint management was associated to improved outcomes. These control groups were extracted from the National Register of Cases of Tuberculosis in Mexico—where all diagnosed cases in the country are mandatorily reported according to the current official guidelines [18]. The first comparison group was a historical control which included 139 patients with TB and DM diagnosed and treated in the same primary care units during the 36 months previous to the present study from a total of 653 pulmonary tuberculosis patients ≥20 years of age. We also compared treatment outcomes of our study group with a same-period control group which included 232 patients with TB and DM diagnosed and treated in the same municipalities but in different primary care units as our study group from a total of 1058 pulmonary tuberculosis patients ≥20 years of age. The demographic, clinical and treatment outcomes of patients with TB and DM from both groups were compared with those of our study population. With 95 subjects in the joint management group and 139 in the comparison group, the study would be able to show an increase of 15% in success rate from an estimated 75% registered for participating health jurisdictions in 2010, with 80% power, at a 5% significance level. The association between treatment success and study group was evaluated using logistic regression analysis after adjusting for sex, age and previous TB treatment. The logistic regression models were validated by evaluating the goodness of fit, model specificity and multicollinearity. As with regression models, variables included in the models were those with *p*-values ≤0.20 in bivariate analysis or with biological plausibility and covariates were arrived to using hierarchical backward elimination approach. All analyses were performed using the STATA 13.0 statistical software package (StataCorp LP, College Station, TX, USA).

### Ethical approval

This study was approved by the Ethical Commission of the Instituto Nacional de Salud Pública (approval number 422). Participants gave written informed consent before taking part. All patients were referred to health facilities to receive treatment in accordance with the stipulations of the National Program for the Prevention and Control of TB and the National Program for the Prevention and Control of DM.

## Results

**Figure 1:**
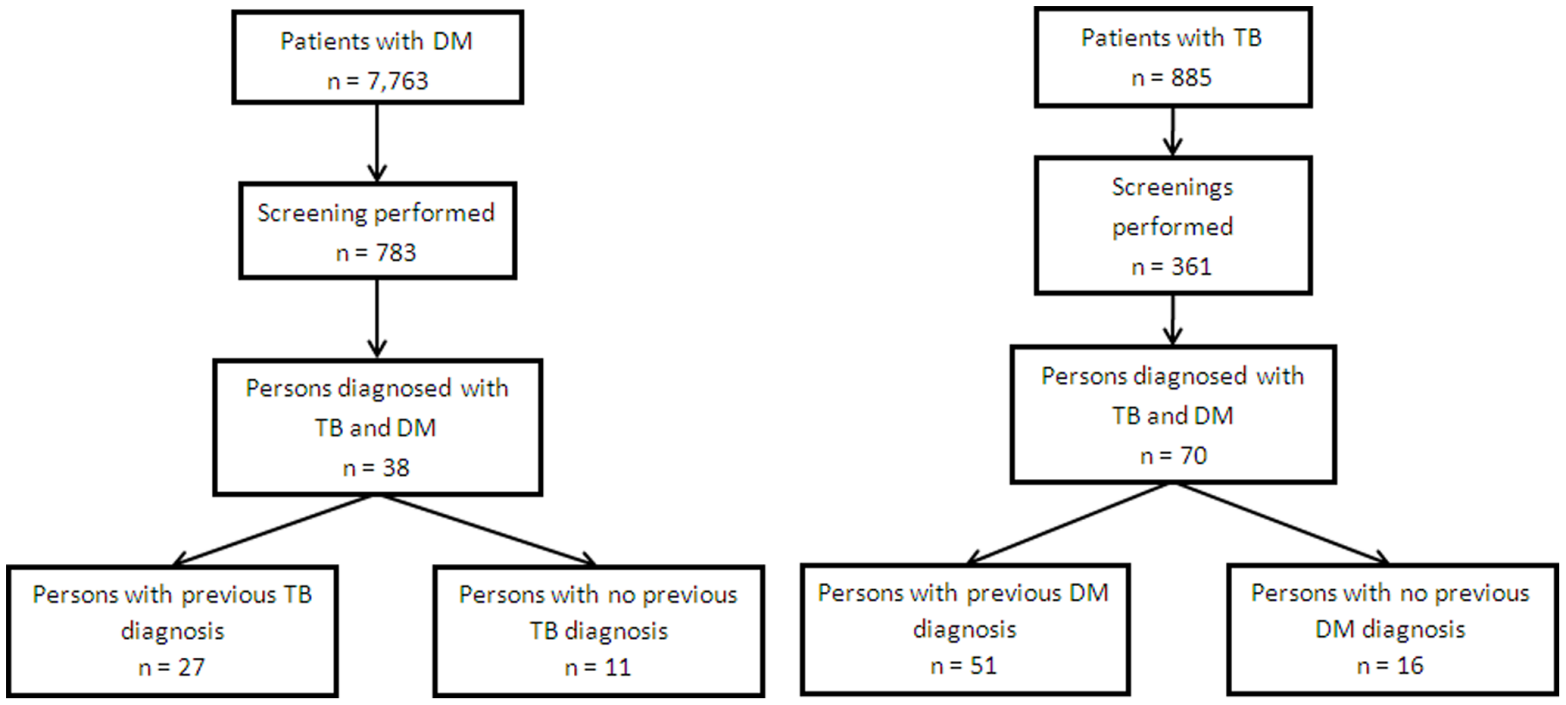
Flowchart of the bidirectional screening for TB and DM (Tijuana, Ciudad Juarez, Reynosa, Guadalupe and Zapopan, Mexico, 2013).

TB screening tests were performed in 783 (10.1%) of the 7,763 subjects with previous DM diagnoses who were listed during the study period at the primary care units. A large proportion of patients were not screened since DM care providers referred that they were too busy to conduct TB symptom screen for all DM cases. Among screened patients, TB was diagnosed in 38 (4.9%), of whom 11 (28.9%) had been unaware of their TB statuses. DM screening was performed in 361 (40.8%) of the 885 patients with recorded bacteriological TB diagnoses at the same clinics during the study period. TB clinic overload and lack of reagents to perform glucose tests explains why not all TB patients were screened for DM. Among these patients, DM was diagnosed in 70 (19.4%), of whom 16 (22.9%) had been unaware of their DM statuses (Figure 1). When we compared TB patients who were screened with those that were not, we found that screened patients were younger (median 38 years [interquartile range, IQR 28–50] versus 41 years [IQR 29.5–55], p = 0.006) but similar regarding the proportion of men and women (males, 64.5% [233/361] versus 70.4% [369/524], p = 0.065).

### TB screening in DM patients

Table 1 shows the characteristics of DM patients in whom pulmonary TB was diagnosed in comparison to those in whom a TB diagnosis was ruled out. Patients who were diagnosed with TB were more likely to be male, thinner, receive treatment with insulin (and less likely to be treated with oral hypoglycaemic agents), have had a previous episode of TB and have had contact with a TB patient. The most frequent symptom was cough with phlegm. Six of 38 patients who did not refer this symptom were patients who had already been diagnosed with TB and were under treatment. Municipalities varied in the proportion of patients detected with TB.

**Table 1:** Characteristics of the DM patients who were screened for TB (Tijuana, Ciudad Juarez, Reynosa, Guadalupe and Zapopan, Mexico, 2013).

Characteristic	Total	Patients with TB	Patients without TB	p-value*
	Number/Total	Number/Total	Number/Total	
	(%)	(%)	(%)	
Male	205/783	20/38	185/745	<0.001
	(26.2)	(52.6)	(24.8)	
Age (years) [median (IQR)]	45	46	49	0.555**
	(57–66)	(56–66)	(57–66)	
***Municipality***				
Tijuana	90/783	1/38	89/745	0.026
	(11.5)	(2.6)	(11.9)	
Ciudad Juarez	349/783	23/38	326/745	
	(44.6)	(60.5)	(43.8)	
Reynosa	49/783	1/38	48/745	
	(6.3)	(2.6)	(6.4)	
Guadalupe	94/783	8/38	86/745	
	(12.0)	(21.1)	(11.5)	
Zapopan	201/783	5/38	196/745	
	(25.7)	(13.2)	(26.3)	
Oral hypoglycaemic agents	568/764	17/37	551/727	<0.001
	(74.4)	(46.0)	(75.8)	
Insulin	48/746	12/37	36/709	<0.001
	(6.4)	(32.4)	(5.08)	
***TB history***				
History of a previous TB episode	25/782	6/38	19/744	<0.001
	(3.2)	(15.8)	(2.6)	
Contact with a TB patient	55/779	8/38	47/741	0.001
	(7.1)	(21.1)	(6.3)	
***Signs and symptoms of TB***				
Fever	57/781	20/37	37/744	<0.001
	(7.3)	(54.1)	(5.0)	
Cough with phlegm	148/782	32/38	116/744	<0.001
	(18.9)	(84.2)	(15.6)	
Weight loss	74/779	21/38	53/741	<0.001
	(9.5)	(55.3)	(7.2)	
Night sweats	109/783	23/38	86/745	<0.001
	(13.9)	(60.5)	(11.5)	
***Anthropometric measures***				
BMI (m/kg^2^) [median (IQR)]	29.3	24.8	29.6	<0.001**
	(25.9–33.9)	(22.5–26.7)	(26.0–34.0)	
Waist circumference (cm) [median (IQR)]	99	88	99	<0.001**
	(90–107)	(81–98)	(91–108)	
Abdominal obesity	573/777	17/37	556/740	<0.001
	(73.7)	(45.9)	(75.1)	

*Chi-squared test;

**U Mann-Whitney test; TB, Tuberculosis; DM, Diabetes mellitus; IQR, interquartile range; BMI, Body mass index.

### DM screening in TB patients

Table 2 lists the characteristics of TB patients in whom DM was diagnosed in comparison to those who were not diagnosed with DM. Patients diagnosed with DM were more likely to be older; have parents with DM; have polydipsia or polyuria and although heavier, were more likely to have recently experienced weight loss. Municipalities varied in the proportion of patients detected with DM.

**Table 2:** Characteristics of TB patients who were screened for DM (Tijuana, Ciudad Juarez, Reynosa, Guadalupe and Zapopan, Mexico, 2013).

Characteristic	Total	Patients with DM	Patients without DM	p-value*
	Number/Total	Number/Total	Number/Total	
	(%)	(%)	(%)	
Male	233/361	39/70	194/291	0.085
	(64.5)	(55.7)	(66.7)	
Age (years) [median (IQR)]	38	45	36	<0.001**
	(28–50)	(35–55)	(26–48)	
***Municipality***				
* Tijuana*	39/361	16/70	23/291	<0.001
	(10.8)	(22.9)	(7.9)	
* Ciudad Juarez*	118/361	3/70	115/291	
	(32.7)	(4.3)	(39.5)	
* Reynosa*	76/361	40/70	36/291	
	(21.1)	(57.1)	(12.4)	
* Guadalupe*	107/361	8/70	99/291	
	(29.6)	(11.4)	(34.0)	
* Zapopan*	21/361	3/70	18/291	
	(5.8)	(4.3)	(6.2)	
***History and risk factors for DM***				
Siblings with DM	89/353	17/66	72/287	0.91
	(25.2)	(25.8)	(25.1)	
Parents with DM	131/354	38/66	93/288	<0.001
	(37.0)	(57.6)	(32.3)	
Women with children weighing ≤4 kg at birth	40/127	12/32	28/95	0.398
	(31.5)	(37.5)	(29.5)	
***Signs and symptoms of DM***				
Polyuria	130/356	37/68	93/288	0.001
	(36.5)	(54.4)	(32.3)	
Polydipsia	137/357	44/68	93/289	<0.001
	(38.4)	(64.7)	(32.2)	
Polyphagia	138/354	27/67	111/287	0.806
	(39.0)	(40.3)	(38.7)	
Weight loss in the last 2 months	188/355	54/68	134/287	<0.001
	(53.0)	(79.4)	(46.7)	
***Anthropometric measures***				
BMI (m/kg^2^) [median (IQR)]	21.9	23.8	21.5	0.002**
	(19.9–25.1)	(20.5–26.1)	(18.9–24.6)	
Waist circumference (cm) [median (IQR)]	71	73	70	0.123**
	(80–88)	(83–91)	(80–87)	
Abdominal obesity	102/358	26/69	76/289	0.06
	(28.5)	(37.7)	(26.3)	

* Chi-squared test;

**U Mann-Whitney test; TB, Tuberculosis, DM, Diabetes mellitus; IQR, interquartile range; BMI, Body mass index.

### Patients with TB and DM who received TB treatment

**Figure 2:**
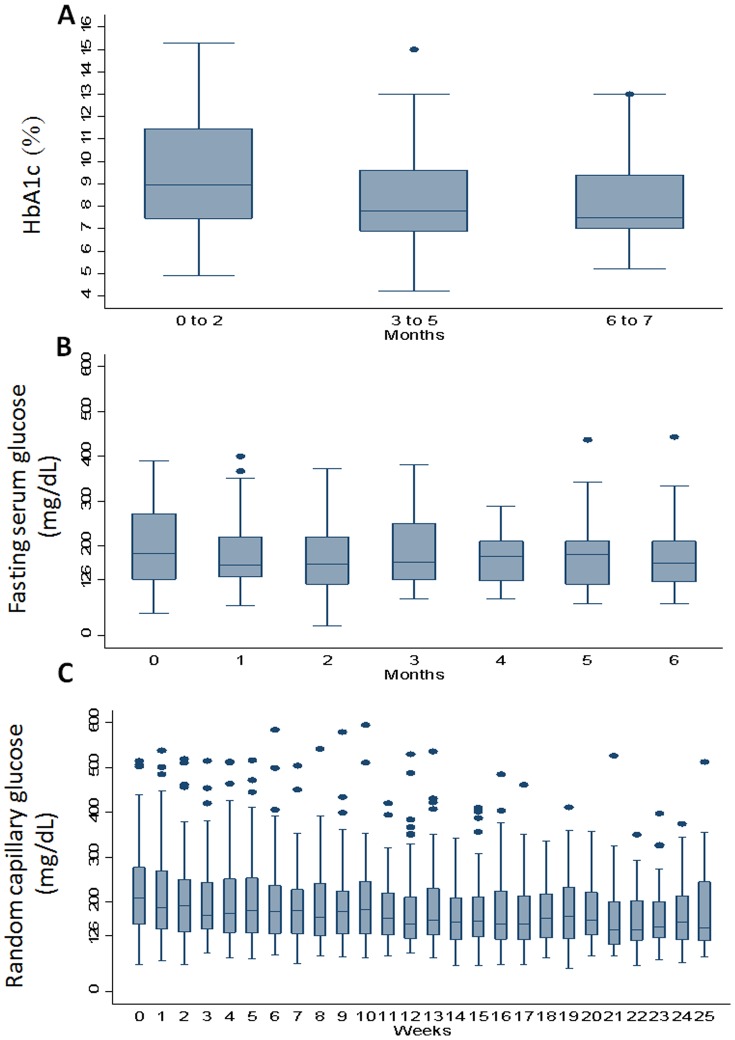
Medians +95% Confidence intervals of measures of glycaemia at each visit among patients with TB and DM during TB treatment (Tijuana, Ciudad Juarez, Reynosa, Guadalupe and Zapopan, Mexico, 2012–2013. Panel A, HbA1c (%); Panel B, Fasting serum glucose (mg/dL); Panel C, Capillary random blood glucose (mg/dL). As TB treatment progressed, the levels of HbA1c and random capillary glucose showed a significant decreasing trend, (regression coefficients (β) −0.660, (95% confidence interval (CI), −0.96 to −0.35); and β = −1.889 (95% CI −2.77 to −1.01, respectively) adjusting by sex, age and having had treatment for a previous TB episode.

Of the 108 patients diagnosed with TB/DM, 87.9% (95/108) agreed to receive treatment under joint management conditions. Table 3 lists the sociodemographic and clinical characteristics of these patients. Eighty one patients (85.26%) were cured; 4 (4.21%) completed treatment; 2 (2.11%) defaulted; 5 (5.26%) failed; 3 (3.2%) died; and none were transferred out (Table 3). By bivariate analyses, patients who successfully completed treatment were more likely to have access to social security (Table 3). Seventy nine patients (81.4%); 65 (67%) and 54 (55.6%) maintained their scheduled appointments for measurement of random capillary glucose, fasting glucose, and HbA1c respectively. As the TB treatment progressed, the levels of HbA1c and random capillary glucose showed a significant decreasing trend, (*p*<0.001; Figure 2). Multiple linear regression analysis with change in HbA1c and random capillary glucose as dependent variables revealed significant decrease with time (p<0.001) adjusting by sex, age and having had treatment for a previous TB episode (Table 4).

**Table 3:** Characteristics of patients with TB and DM (Tijuana, Ciudad Juarez, Reynosa, Guadalupe and Zapopan, Mexico, 2013).

Characteristics	Total	Treatment success	Death, Failure or Default	*p-value* †
	Number/Total	Number/Total	Number/Total	
	(%)	(%)	(%)	
Male	49/95	41/85	8/10	0.057
	(51.6)	(48.2)	(80.0)	
Age (years) [median (IQR)]	50	50	50.5	0.836¶
	(39–58)	(39–58)	(41–65)	
***Municipality***				
Tijuana	13/95	8/85	5/10	0.007
	(13.7)	(9.4)	(50.0)	
Ciudad Juarez	26/95	23/85	3/10	
	(27.4)	(27.1)	(30.0)	
Reynosa	36/95	34/85	2/10	
	(37.9)	(40.0)	(20.0)	
Guadalupe	16/95	16/85	0/10	
	(16.8)	(18.8)	(0.0)	
Zapopan	4/95	4/85	0/10	
	(4.2)	(4.7)	(0.0)	
Social Security	80/95	74/85	6/10	0.026
	(84.2)	(87.1)	(60.0)	
<6 years of schooling	48/91	42/81	6/10	0.626
	(52.7)	(51.9)	(60.0)	
>10 drinks per week	6/95	5/85	1/10	0.613
	(6.3)	(5.9)	(10.0)	
>10 cigarettes per week	3/95	3/85	0/10	0.546
	(3.2)	(3.5)	(0.0)	
Time since DM diagnosis, (years) [median (IQR)]; n = 95	3	3	4.5	0.196¶
	(0–12)	(0–11)	(2–17)	
At least 1 complication of diabetes‡	21/95	19/85	2/10	0.865
	(22.1)	(22.4)	(20.0)	
Comorbidities	3/95	3/85	0/10	0.546
	(3.2)	(3.5)	(0.0)	
Resistance to isoniazid and rifampicin	1/87	1/79	0/8	0.749
	(1.1)	(1.3)	(0.0)	
Previous TB treatment	13/94	10/84	3/10	0.117
	(13.83)	(11.90)	(30.00)	
Haemoptysis	28/92	26/84	2/8	0.727
	(30.4)	(31.0)	(25.0)	
Fever	56/90	50/82	6/8	0.435
	(62.2)	(61.0)	(75.0)	
BMI (kg/m^2^) [median (IQR)]	24.33	24.71	23.05	0.459¶
	(21.51–27.81)	(21.51–27.81)	(22.13–26.15)	

† Chi-squared test.

‡ At least one of the following complications: retinopathy, hypertension, renal disease, renal failure, diabetic foot, obesity, neuropathy (mono or polyneuropathy), visceral neuropathy (diarrhoea, erectile dysfunction, etc.), urinary albumin, chest pain or other.

¶ Mann–Whitney test. TB, Tuberculosis; DM, Diabetes mellitus; IQR, interquartile range; BMI, Body mass index.

**Table 4:** Robust random effects linear regression for longitudinal data with change in HbA1c, fasting serum glucose and random capillary glucose as dependent variable among patients with TB and DM (Tijuana, Ciudad Juarez, Reynosa, Guadalupe and Zapopan, Mexico, 2013).

Characteristics	HbA1c (%)		Fasting serum glucose (mg/dL)		Random capillary glucose (mg/dL)	
	Beta regression coefficients		Beta regression coefficients		Beta regression coefficients	
	IC95%		IC95%		IC95%	
	All patients	Treatment success	All patients	Treatment success	All patients	Treatment success
	n = 88	n = 80	n = 75	n = 71	n = 94	n = 84
Time to treatment completion	−0.660**	−0.711**	−3.510*	−3.357	−1.889**	−1.942**
	[−0.96, −0.35]	[−0.98, −0.43]	[−7.56,0.55]	[−7.53,0.81]	[−2.77, −1.01]	[−2.85, −1.03]
Men	0.165	0.147	−17.73	−16.72	−6.728	−11.13
	[−0.77, 1.10]	[−0.82,1.12]	[−46.67,11.20]	[−46.10,12.65]	[−6.25,27.79]	[−41.68,19.42]
Age (years)	−0.0321	−0.033	−1.192*	−1.099	−0.589	−0.255
	[−0.06,0.003]	[−0.06,0.002]	[−2.34, −0.04]	[−2.27,0.07]	[−1.67,0.49]	[−1.26,0.75]
Previous TB treatment	0.413	0.362	16.06	27.51	14.35	23.62
	[−1.02,1.84]	[−0.78,1.50]	[−30.49,62.61]	[−24.28,79.30]	[−22.65,51.35]	[−18.65,65.88]

TB =  Tuberculosis; DM =  Diabetes mellitus; 95% CI = 95% Confidence interval;

*p<0.05,

**p<0.001.

One hundred thirty nine patients were diagnosed and treated between June 2009 and June 2012 in participating clinics and 232 patients were treated between July 2012 and April 2013 in the same municipalities but different clinics. Patients under joint management were more likely to cure and less likely to complete treatment without showing evidence of treatment failure and without negative bacteriology and to default as compared to both control groups. No statistically significant differences were observed between the study group and the control groups regarding sex, age and previous TB treatment (Table 5).

**Table 5:** Comparison of the sociodemographic characteristics and treatment outcome of patients with TB/DM diagnosed and treated in the same clinics from June 2009 to June 2012 (historical control) and in the same municipalities but different clinics from July 2012 to April 2013 (same period control), as compared to study population (Tijuana, Ciudad Juarez, Reynosa, Guadalupe and Zapopan, Mexico).

Variable	Study population	Historical control	p value* ‡	Same period control	p value* ‡
	Number/Total	Number/Total		Number/Total	
	(%)	(%)		(%)	
	n = 95	n = 139		n = 232	
Male	49/95	86/139	0.118	142/232	0.109
	(51.58)	(61.87)		(61.21)	
Age (years) [median (IQR)]	50	52	0.973†	52	0.402†
	(39–58)	(41–58)		(41–61)	
Previous TB treatment	13/94	26/139	0.328	28/232	0.664
	(13.83)	(18.71)		(12.07)	
Treatment success	85/95	98/132	0.004	179/228	0.020
	(89.47)	(74.24)		(78.51)	
Cure	81/95	78/132	<0.001**	128/228	<0.001**
	(85.26)	(59.09)		(56.14)	
Treatment completion	4/95	20/132	<0.001**	51/228	<0.001**
	(4.21)	(15.15)		(22.37)	
Default	2/95	22/132	0.008**	17/228	0.063**
	(2.11)	(16.67)		(7.46)	
Failure	5/95	4/132	0.395**	9/228	0.597**
	(5.26)	(3.03)		(3.95)	
Death	3/95	6/132	0.597**	21/228	0.059**
	(3.16)	(4.55)		(9.21)	
Transfer out	0/95	2/132	0.228**	2/228	0.360**
	(0.00)	(1.52)		(0.88)	

*Pearson's chi-squared test;

**Binomial test;

† Mann-Whitney U test;

‡ As compared to study population. TB = Tuberculosis; DM = Diabetes mellitus; IQR = Interquartile range.

As shown in Tables 5 and 6, by bivariate and multivariate analyses, patients who were treated according to the pilot model of joint management were more likely to experience treatment success than were those who had been treated in the same clinical units during the previous 36 months or those treated in the same municipalities but in different clinics during the same period as the study group after adjusting for sex, age and the previous TB treatment history.

**Table 6:** Variables associated with treatment success in patients with TB/DM who were treated via joint management in the same clinics from June 2009 to June 2012 (historical control, Model 1) and in the same municipalities but different clinics from July 2012 to April 2013 (same period control, Model 2), as compared to study population (Tijuana, Ciudad Juarez, Reynosa, Guadalupe and Zapopan, Mexico).

Variables	Model 1	P value*	Model 2	P value*
	aOR		aOR	
	(95% CI)		(95% CI)	
	n = 226		n = 322	
Patients treated under the joint management model *versus* patients in control group	2.80	0.010	2.37	0.022
	(1.28–6.13)		(1.13–4.96)	
Age (years)	1.01	0.235	1.00	0.808
	(0.98–1.04)		(0.98–1.02)	
Male	0.56	0.130	0.95	0.877
	(0.26–1.18)		(0.52–1.74)	
Previous TB treatment	0.31	0.005	0.40	0.020
	(0.13–0.70)		(0.18–0.86)	

*Logistic regression analysis TB =  Tuberculosis; DM = Diabetes mellitus; aOR =  Adjusted Odds ratio; 95% CI = 95% confidence interval.

## Discussion

Our study demonstrates the feasibility of implementing a joint management model for the TB-DM association in Mexico and provides preliminary evidence of its effectiveness to improve treatment outcomes. Among the screenings conducted in DM patients, 11 (1.38%) had been previously unaware of their TB status, whereas in screenings conducted in TB patients, 16 (4.4%) had been unaware of their DM status, thus demonstrating the benefit of the strategy in this setting. Accordingly, 71 people with DM would need to undergo screening to detect a new case of TB, whereas only 22 people with TB would need to undergo screening to detect a new case of DM. On the other hand, the glucose measurements were found to significantly decrease during treatment in 95 recruited patients with both comorbidities. Finally, the proportion of patients who successfully completed treatment under the joint management model was higher than the success rate achieved at the same units during the previous 36 months and at different primary care clinics in the same municipalities during the same study period.

Prevalence of TB among patients with DM in a recent systematic review ranged from 1,995 to 36,206 per 100,000 individuals with DM. [8] TB prevalence among DM patients in our study (4,853 per 100,000 DM patients) is comparable to some of these studies (e.g. Tripathy in India in 1984) although higher than reports of recent screenings conducted in India and China (705 and 774 per 100, 000 DM patients). [10], [19] TB prevalence among DM patients was 147-fold higher than WHO reported prevalence for general population in Mexico, (33 (2.5th and 97.5th centiles, 16–57) per 100,000 inhabitants in 2012. [1] This notoriously higher TB prevalence among DM patients suggests that TB transmission may be occurring in health care settings in Mexico. This is supported by previous reports on TB transmission to health workers. [20] Moreover, usage of molecular tools has previously documented the occurrence of exogenous reinfection in one-fifth of the DM patients in a study conducted in Southern Mexico, suggesting that nosocomial TB transmission might be occurring as a result of DM patients attending clinics where there is a high prevalence of diagnosed and undiagnosed TB. [5] Lack of compliance to international guidelines (inadequate design or renovation of health units and insufficient administrative and environmental controls) to prevent TB transmission in both primary care centres and specialised units would favour this transmission. [21]

The number of individuals with diabetes who would need to undergo screening to detect a new TB case has ranged from four in Sweden in 1961 to 442 in Korea in 1995, and 236 to 1036 in China and India in 2012; [8]–[10], [19], [22] therefore, the number detected in our study (n = 71) confirms that the number of individuals largely depends on the prevalence of both diseases in the region studied.

Prevalence of DM among patients with TB in our study (19.4%) was intermediate as compared to Jeon and colleagues' recent systematic review (3.5% to 35.2%) [8]. The DM frequency among TB patients was two-fold higher than self-reported DM prevalence (9.17%, 95% confidence interval, 8.79%–9.54%) reported for Mexican individuals >20 years of age in 2012 by a probabilistic, cluster household survey interviewing 46,303 individuals >20 years old conducted by the Mexican Secretariat of Health [23], although this figure could be higher due to the proportion of adults who are unaware of their condition. Our results indicate that only 23 DM patients would need to undergo screening to detect a new case of TB. This finding is similar to those of recent studies in India, where it was found that 6–34 TB patients would need to undergo screening to detect new cases of DM. [9], [22], [24]–[26]

Comparison of the characteristics between patients in whom TB or DM was detected allowed the identification of a profile of patients among whom screening could be optimised. In DM patients, the probability of a TB diagnosis was higher in men and in patients who had already suffered a previous TB episode or who had contact with a TB patient, characteristics that have been previously described. [19] It would have been very useful to have information on smoking among DM patients given the association between smoking and TB. [27] However, this information was not available. On the other hand, the probability of a DM diagnosis was associated with older age, having parents with DM and exhibiting signs of recent weight loss, polydipsia and polyuria. Of these factors, only age has been previously described. [22], [24]–[26]

Although the design of our study did not allow us to establish causal relationships between joint management and better blood glucose control and improved treatment outcomes, our data suggest that these associations may exist. In our study, a significant proportion (89.47%, 85/95) of patients were cured or completed treatment in 2012–2013; this compared favourably with treatment outcomes achieved during the previous 36 months at the same health units (2.80 (1.28 to 6.13) and during the same study period at different primary care clinics in the same municipalities (2.37 (1.13–4.96). This improved prognosis might be associated with better glucose control since we observed significant decrease of HbA1c and random glucose level as treatment progressed. The negative impact of hyperglycaemia or high HbA1c levels on the immune responses of TB patients has been previously described. [28]–[31] Accordingly, by improving the glucose levels, the immune response would also improve, and a better treatment outcome would be achieved. Nurses as providers of treatment under DOTS probably had an important role in facilitating compliance or giving patient education as has been documented previously. [32]

This proposal was based on the CFTB/DM, which was recently proposed by the WHO and Union. [6] We have demonstrated the feasibility of bidirectional screening as well as joint management. Additionally, we have also identified problems that need to be addressed in the future in order to improve the effectiveness of this approach such as increasing screening coverage and completing scheduled appointments for measurement of random capillary glucose, fasting glucose, and HbA1c. Health care providers found it difficult to add screening activities in busy clinics. Lack of priority given to this program by some health authorities at the state and local levels resulted in frequent staff turnover, lack of reagents, and delay in laboratory reports explaining some of these problems. In addition lack of appropriate infrastructure to prevent TB transmission in specialized units delayed referral of patients while still transmissible.

The strengths of our study include the following: 1) our study was conducted in primary care clinics in a middle income setting with high prevalence of both DM and TB therefore allowing generalizability to similar regions; 2) bacteriological confirmation of TB diagnoses; 3) blood glucose and HbA1c follow up during TB treatment; 4) we were able to adjust for relevant confounders when analysing glucose control and treatment outcomes.

The present study had several limitations. The design of this observational study did not allow the randomisation of participating or control primary care clinics. Therefore, treatment results of this study were compared with those observed at the same clinics during the previous 36 months and at different primary care clinics in the same municipalities during the same study period. Consequently, organisational and human factors unrelated to the joint management of both diseases might partially explain the differences in the cure rates observed in our study. Outcomes are also dependent on DM disease characteristics such as duration of diabetes, glucose control, or type of treatment. This information was not available among our comparison groups; therefore we were unable to compare these parameters with our study population. Diagnoses of TB were based on sputum smears that are recognized with a low sensitivity. [33] Most of the clinics included in the study did not have chest X ray facilities; therefore it would not have been feasible to conduct chest X rays to improve detection rates. TB patients who were screened for DM were found to be younger that patients who were not screened, therefore the frequency of DM among TB patients may be underestimated. A proportion of our patients might have suffered from glucose intolerance, given that some of the patients received a simultaneous diagnosis of both diseases. Finally we did not measure client satisfaction nor assessed cost-effectiveness.

## Conclusions

This report provides preliminary evidence suggesting that bidirectional screening and joint management in primary care settings is feasible. Treatment success of patients jointly managed compared favourably with outcomes achieved under routine DM and TB management during the previous 36 months at the same primary care clinics and in different clinics in the same municipalities during the same period. Given the growing global epidemic of DM, it is necessary to incorporate bidirectional screening and joint management in order to control TB and DM and to evaluate the effectiveness of this approach in controlled trials. The concurrence of both diseases represents a risk to the possible spread of TB worldwide as well as serious implications for TB control and the achievement of the Millennium Development Goals [34].

## Supporting Information

Data set S1 Data set of historical control and study population.
(ZIP)

Data set S2 Data set of same time control and study population.
(XLS)
